# Specialized odorant receptors in social insects that detect cuticular hydrocarbon cues and candidate pheromones

**DOI:** 10.1038/s41467-017-00099-1

**Published:** 2017-08-17

**Authors:** Gregory M. Pask, Jesse D. Slone, Jocelyn G. Millar, Prithwiraj Das, Jardel A. Moreira, Xiaofan Zhou, Jan Bello, Shelley L. Berger, Roberto Bonasio, Claude Desplan, Danny Reinberg, Jürgen Liebig, Laurence J. Zwiebel, Anandasankar Ray

**Affiliations:** 1 Department of Entomology, University of California Riverside, Riverside, CA 92521 USA; 2 Department of Biological Sciences, Vanderbilt University, Nashville, TN 37235 USA; 3 Department of Cell and Developmental Biology, Epigenetics Program, University of Pennsylvania School of Medicine, Philadelphia, PA 19104 USA; 4 Department of Biology, New York University, New York, NY 10003 USA; 5 Department of Biochemistry and Howard Hughes Medical Institute, New York University School of Medicine, New York, NY 10016 USA; 6 School of Life Sciences, Arizona State University, Tempe, AZ 85287 USA; 7 Center for Disease Vector Research, University of California Riverside, Riverside, CA 92521 USA

## Abstract

Eusocial insects use cuticular hydrocarbons as components of pheromones that mediate social behaviours, such as caste and nestmate recognition, and regulation of reproduction. In ants such as *Harpegnathos saltator*, the queen produces a pheromone which suppresses the development of workers’ ovaries and if she is removed, workers can transition to a reproductive state known as gamergate. Here we functionally characterize a subfamily of odorant receptors (Ors) with a nine-exon gene structure that have undergone a massive expansion in ants and other eusocial insects. We deorphanize 22 representative members and find they can detect cuticular hydrocarbons from different ant castes, with one (HsOr263) that responds strongly to gamergate extract and a candidate queen pheromone component. After systematic testing with a diverse panel of hydrocarbons, we find that most *Harpegnathos saltator* Ors are narrowly tuned, suggesting that several receptors must contribute to detection and discrimination of different cuticular hydrocarbons important in mediating eusocial behaviour.

## Abstract (web summary)

Cuticular hydrocarbons (CHC) mediate the interactions between individuals in eusocial insects, but the sensory receptors for CHCs are unclear. Here the authors show that in ants such as *H. saltator*, the 9-exon subfamily of odorant receptors (HsOrs) responds to CHCs, and ectopic expression of *HsOrs* in *Drosophila* neurons imparts responsiveness to CHCs.

## Introduction

In eusocial insects, interactions between individuals are mediated by cuticular hydrocarbons (CHCs) and other cues which mediate major aspects of colony organization^1–10^, such as nestmate and caste recognition, reproductive division of labour, task recognition, and worker policing^1–4, 11^. In particular, saturated hydrocarbons have been shown to act as queen pheromones in several hymenopterans by repressing development of ovaries in workers^12^. In the ponerine ant *Harpegnathos saltator*, queens and workers display distinct CHC profiles, where queens produce longer chain CHCs^13^. When an *H. saltator* queen senesces or is removed, a subset of workers engage in duelling behaviour, after which a few individuals transition into egg-laying reproductives called gamergates^5^. As an *H. saltator* worker transitions to a gamergate, a distinct shift toward longer-chain compounds is observed in her CHCs, which alters the behaviour of other workers towards her^13–15^. The newly established gamergate is treated as a reproductive and is not targeted for policing, in contrast to those workers which begin to develop ovaries in the presence of a queen or previously established gamergates^16^.

Ants are also able to discriminate differences in the CHC profiles of nestmates and non-nestmates^1–3^. This can occur in the absence of physical contact, suggesting that CHCs can act as olfactory signals in close proximity^17, 18^. Indeed, CHCs are detected by the antennae, as demonstrated by antennal electrophysiology and calcium imaging from antennal lobes in various ant species stimulated with CHCs^19–23^. However, the olfactory receptors for CHC detection have not been previously identified. Identification and functional characterization of CHC receptor genes can provide insight into the complex chemical communication systems utilized by eusocial insects.

Transcriptome profile analysis of antennae from *H. saltator* has identified several possible chemoreceptor families that are expressed^6^. Of these, the odorant receptor (*Or*) family exhibits a large expansion, with 347 putative *Harpegnathos saltator Ors* (*HsOrs*). A third of *HsOrs* are more highly expressed in antennae of workers compared to males, with most of these receptors belonging to a distinct nine-exon subfamily^6^. The nine-exon subfamily is also greatly expanded in eusocial insects, and constitutes approximately one-third of the total *Or* family in annotated ant genomes^6–10^. With such a rapid rate of evolution and sexually dimorphic expression, the nine-exon *Or* subfamily represent likely candidates for CHC receptors.

Through the use of transgenic expression of *HsOrs* in *Drosophila melanogaster*, we have functionally characterized 22 representative HsOrs from the nine-exon subfamily as CHC detectors and demonstrate that most HsOrs are narrowly tuned to specific hydrocarbon structures. A highly-expressed *HsOr* in workers, *HsOr263*, was identified as a detector of a candidate reproductive signal due to its strong responses to both gamergate extract and a predicted queen pheromone component. Together, these studies establish the nine-exon subfamily of Ors as CHC detectors that are likely critical for proper organization of eusocial insect colonies.

## Results

### Functional expression of *HsOrs* in *Drosophila*

**Fig. 1:**
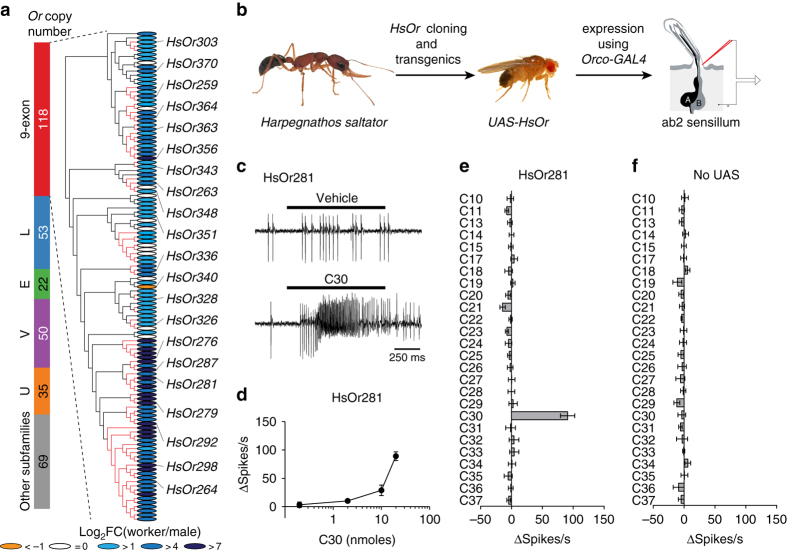
Functional identification of HsOrs that act as receptors for cuticular hydrocarbons. **a** Phylogeny of the nine-exon subfamily of HsOrs (*left*) and the HsOrs cloned and characterized in this study (*right*). *H. saltator* specific branches are in *red* and *coloured ovals* indicate the relative enrichment in worker to male comparisons for each nine-exon HsOr. **b** Schematic for functional characterization of HsOrs in *D. melanogaster*. **c** Representative traces of recordings from an ab2 sensillum of a *w* ^*+*^, *UAS-HsOr281; w* ^*+*^, *Orco-GAL4* fly in response to the heated control cartridge (pentane solvent only, *top*) and 20 nmol of triacontane (C30, *bottom*). **d** Dose-dependent responses from HsOr281-expressing neurons to different doses of C30 (*n* = 5). **e**, **f** Responses of the ab2A neuron in *w* ^*+*^, *UAS-HsOr281; w* ^*+*^ *, Orco-GAL4* flies **e** and +; *w* ^*+*^ *, Orco-GAL4* flies **f** to a hydrocarbon panel. All compounds were applied at 20 nmol and each value represents mean ± SEM (*n* = 6)

To examine the function of individual members of the nine-exon *HsOrs*, we analysed transcriptomic and phylogenetic data to identify *HsOr* transcripts that were relatively more abundant in antennae of workers compared to males, and were conserved across different evolutionary branches of the subfamily. Of the 22 *HsOrs* selected, all but one were significantly enriched in antennae of workers compared to those of males, whereas *HsOr340* was male-enriched (Fig. 1a)^6^. To identify ligands for these receptors, we utilized a functional expression strategy in *D. melanogaster* neurons, generating *UAS-HsOr* transgenic fly lines for each of the 22 *HsOr* genes, to be used together with previously established *GAL4* driver lines.

We expressed our first candidate CHC receptor, *UAS-HsOr281*, in the majority of olfactory receptor neurons (ORNs) of *D. melanogaster* using the strong *Orco-GAL4* driver and performed single-sensillum electrophysiology on the ab2 antennal sensilla to measure responses to a panel of alkanes (Fig. 1b–e). As done in previous studies, we heated the delivery cartridge for ~1 s (112.5 ± 2.8 °C, *n* = 10) with a butane torch to facilitate the volatilization of the stimulus and applied a 1 s pulse via a humidified airflow over the *HsOr*-expressing fly antenna^20, 23, 24^. In these studies, triacontane (C30), a hydrocarbon present on the *H. saltator* cuticle^13^, elicited robust and dose-dependent responses in HsOr281-expressing ab2A neurons, whereas other alkanes elicited little activity (Fig. 1c–e). In control flies that lacked the *UAS-HsOr281*, the ab2A neuron was indifferent to alkanes (Fig. 1f), demonstrating that a nine-exon subfamily HsOr can indeed function as a highly specific CHC receptor.

### *HsOrs* respond to CHC extracts of different castes

**Fig. 2:**
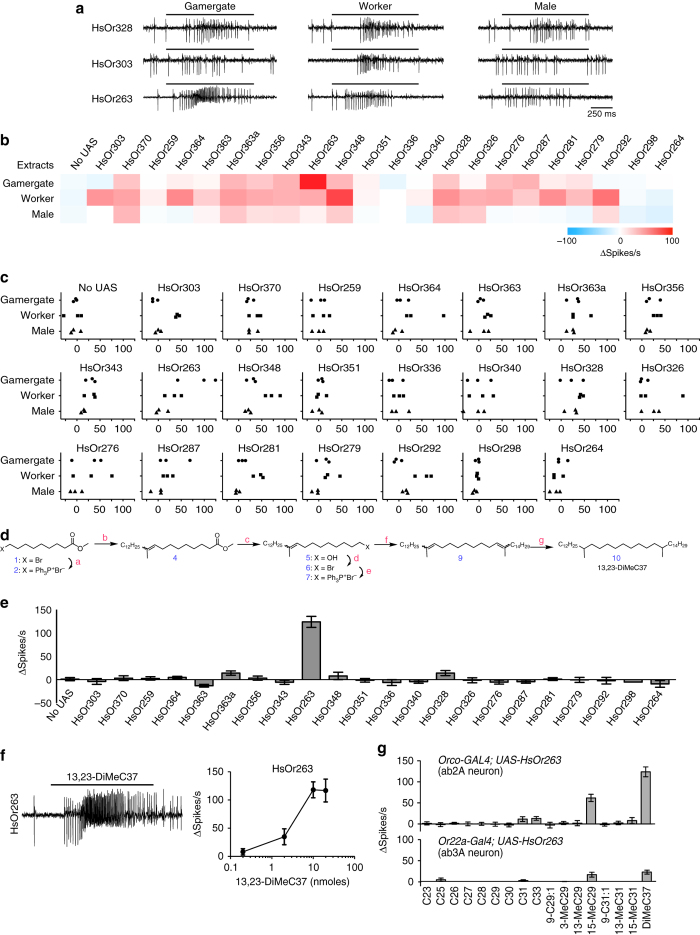
Nine-exon HsOrs detect CHC extracts from different castes and HsOr263 detects a candidate queen pheromone component. **a** Representative action potential traces of three HsOrs to cuticular extracts of gamergates (*left*), workers (*middle*), and males (*right*). **b** Heat map of mean HsOr responses to each *H. saltator* extract. All extracts were applied at 1.2 µg of total hydrocarbon, and *n* = 3 for each extract-HsOr combination. **c** Responses to each of the 3 individual extracts for each caste for each HsOr tested. **d** Schematic of synthesis of 13,23-DiMeC37. (a) Ph_3_P, acetonitrile, reflux; (b) NaHMDS, then 2-tetradecanone **3**, CH_2_Cl_2_; (c) LiAlH_4_, THF; (d) Ph_3_P, CBr_4_, CH_2_Cl_2_; (e) Ph_3_P, 110 °C; (f) NaHMDS, then 2-hexadecanone, CH_2_Cl_2_; (g) 5% Pd/C, H_2_, hexane. 13,23-DiMe37 is an ~equal mixture of the four stereoisomers. **e** Mean electrophysiological responses of the different HsOrs to 20 nmol of racemic 13,23-DiMeC37 (*n* = 6, error = s.e.m.). **f** A representative excitatory response to 13,23-DiMeC37 from an ab2 sensillum expressing HsOr263 and dose-dependent responses to different amounts of 13,23-DiMeC37 (*n* = 5). **g** Mean responses of HsOr263 to a panel of hydrocarbons found on *H. saltator* cuticle when expressed using *Orco-GAL4* (*w* ^*+*^ *, UAS-HsOr263; w* ^*+*^ *, Orco-GAL4*, ab2A neuron, *n* = 6, *top*) or the empty neuron (*Δhalo*; *w* ^*+*^ *, Or22a-GAL4/ w* ^*+*^ *, UAS-HsOr263*, ab3A neuron, *n* = 5, *bottom*). All methyl-branched hydrocarbons used here and in subsequent experiments were racemic mixtures unless otherwise stated. (Error = SEM)

One of the key functions of the ant olfactory system is to inform an individual about the caste and reproductive status of colony members. To determine whether nine-exon *Ors* contribute to detecting and discriminating between males, non-reproductive workers, and gamergates, we systematically measured responses of each of the 22 HsOrs to CHC extracts derived from these types of individuals. CHC extracts were prepared from individuals and analysed by gas chromatography mass spectrometry (GC/MS) for quality control before pooling together CHC extracts from males, gamergates, and workers, respectively. While the majority of the nine-exon receptors responded to one or more of the extracts, a subset, such as HsOr328, responded to all three, whereas others, for example HsOr303, responded primarily to extracts of workers (Fig. 2a–c). HsOr263 showed significantly greater responses to gamergate extracts, suggesting that it detects a pheromone that is present and possibly abundant in gamergates (Fig. 2a–c).

### An *HsOr* detects a candidate queen pheromone component

A previous study had identified 13,23-dimethylheptatriacontane (13,23-DiMeC37), as a candidate component of the *H. saltator* queen pheromone that signals her fertility to workers^13^. This compound was present mainly on reproductive queens and gamergates, where it comprised nearly 5% of the total CHCs^13^. To identify its molecular receptor, we synthesized racemic 13,23-DiMeC37 (Fig. 2d) and systematically tested it against all 22 HsOrs (Fig. 2e). It elicited a strong response only from HsOr263. This suggests that 13,23-DiMeC37 is an active component of the strong response of HsOr263 to gamergate extract (Fig. 2e, f). We further explored the response profile of HsOr263 to a panel of hydrocarbons found in *H. saltator* cuticular extracts^13^. The panel consisted of commercially available alkanes, and other synthesized CHCs with double bonds and methyl branches in various positions (see Methods). These studies revealed that 15-methylnonacosane (15-MeC29), a compound present in both worker and gamergate cuticular extracts^13^, also activated HsOr263, although to a lesser extent (Fig. 2g). We also expressed HsOr263 in the ab3A sensilla using the classical “empty neuron” technique and the *Or22a-GAL4* driver (Fig. 2g)^25^. A similar HsOr263 response profile was observed within the empty neuron, although lower in magnitude than with *Orco-GAL4*, consistent with expectations from a weaker expressing GAL4 driver (Fig. 2f). A linear regression analysis showed a very good correlation (*R* ^2^ = 0.924) between the HrOr263 hydrocarbon responses in the ab3A and ab2A neurons, which was highly significant (*p* < 0.0001). Additionally, when responses were normalized to the maximal increase in spike frequency for each dataset, an analysis of variance of the normalized spike responses showed that the effect of the different expression systems (ab3A and ab2A) was not significantly different (*p* = 0.13) for the response profile from the tested hydrocarbons. These results demonstrate that the *H. saltator-*specific, worker-enriched HsOr263 is a receptor for a candidate pheromone component, and suggest this receptor plays an important role in the detection of reproductive individuals within the colony.

### Screening of *HsOrs* to a hydrocarbon panel

**Fig. 3:**
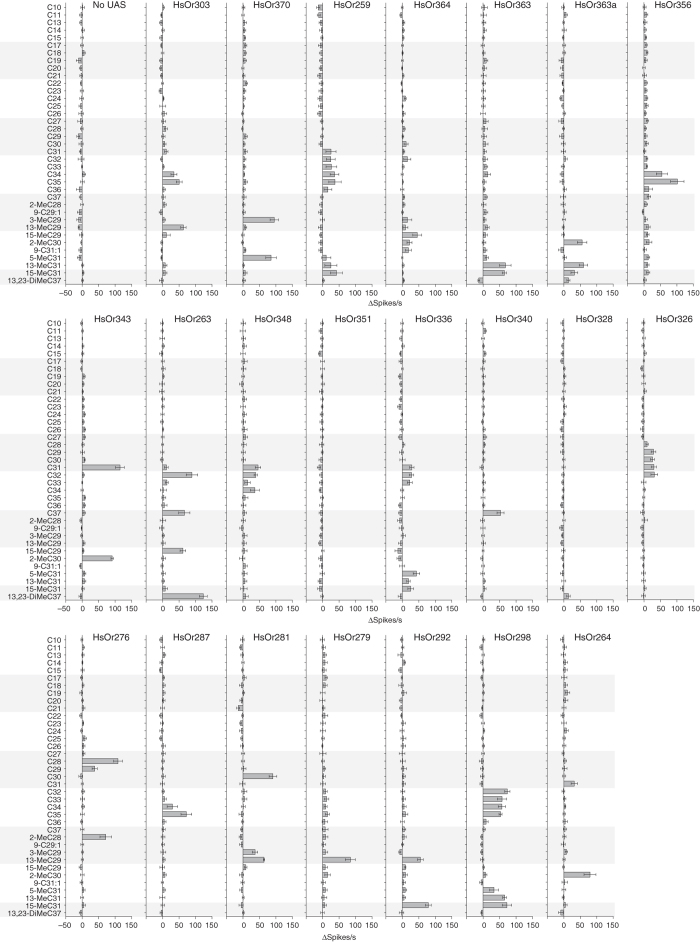
Combinatorial detection of hydrocarbons across the nine-exon HsOrs. Mean responses of each HsOr to a panel of hydrocarbons (each at 20 nmol) from the ab2A neuron of *w* ^*+*^ *, UAS-HsOr; w* ^*+*^ *, Orco-GAL4* flies. Values represent mean ± SEM (*n* = 6)

Because little is known about how odorant receptor proteins might detect this class of low volatility long-chain hydrocarbons, we screened each of the 22 HsOrs with a larger panel of CHCs which comprised a subset of CHCs from *H. saltator*, as well as hydrocarbons which are not present in this species (Fig. 3). Each HsOr displayed a characteristic and narrow response spectrum, where structurally-related hydrocarbons that differed only by a single carbon often resulted in dramatic differences in HsOr activity. For example, 13-MeC29 and 15-MeC29 elicited differential responses across several HsOrs, demonstrating that the position of the methyl group on the carbon chain significantly affected receptor activation. A few receptors exhibited slightly broader response profiles, such as HsOr298, which responded to C32-C35 and the methyl-branched C31 compounds.

**Fig. 4:**
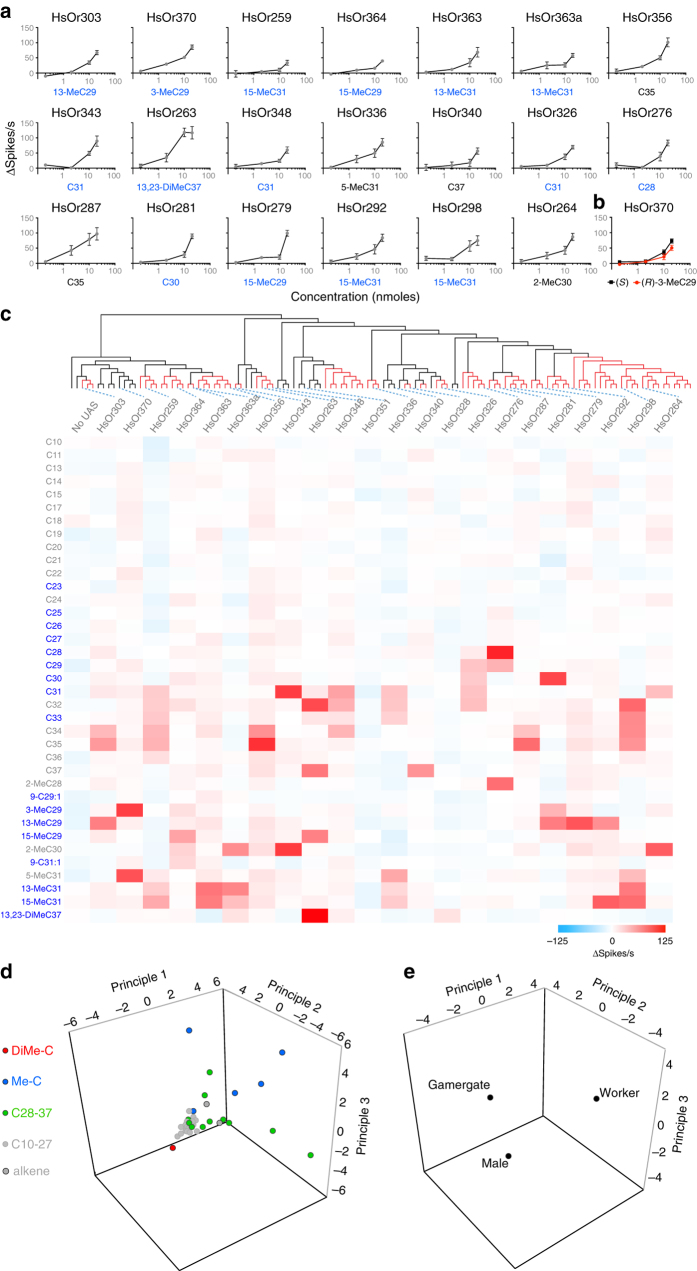
Hydrocarbon coding of the nine-exon subfamily. **a** Dose-response curves for the HsOrs with the best ligands identified earlier. *X*-axes represent nmol of compound and values are mean ± SEM (*n* = 5). **b** Dose-response curve for HsOR370 to (*R*)-3-MeC29 and (*S*)-3-MeC29. *X*-axes represent nmol of compound and values are mean ± SEM (*n* = 5) **c** A heat map of HsOr responses to the entire hydrocarbon panel (each at 20 nmol). Hydrocarbon names in *blue* are present in *H. saltator* cuticular extracts. **d** The first three principal components of a receptor activity-based space of hydrocarbon detection. Hydrocarbons of different classes are colour-coded. **e** The first three principal components of a mean receptor activity-based space of cuticular extracts from males, gamergates, and workers

We also examined two active isoforms of HsOr363 that differed by 14 amino acids. Interestingly, while both responded to 13- MeC31 and 15-MeC31, only the shorter isoform, HsOr363a, responded to 2-MeC30 (Fig. 3). Two receptors (HsOr328 and HsOr351) did not respond to any hydrocarbons in the panel. Nonetheless, HsOr328 responded to all three cuticular extracts, suggesting that its ligand is present in the *H. saltator* cuticle but absent from our test panel. For each receptor, we tested multiple doses of the best hydrocarbon ligand and demonstrated clear dose-dependent responses (Fig. 4a). It had been reported that the chiral methyl-branched CHCs of a number of insect species, including Hymenoptera, typically exist in the (*R*)-configuration^26^. Thus, we tested whether HsOr370, which was strongly activated by racemic 3-MeC29, might respond differentially to the (*R*)- and (*S*)-enantiomers of 3-MeC29. At the doses tested, we found no significant difference between the responses of HsOr370 to either (*S*)- or (*R*)-3-MeC29 across a range of concentrations (Fig. 4b). However, this is only one receptor-ligand pair, and it is possible that other HsOrs could have significant differences in activities between enantiomers. Taken together, these experiments demonstrate that the nine-exon subfamily of *HsOr*s receptors in *H. saltator*, and by extension, other hymenopteran *Or*s, are able to function as detectors of CHCs, many of which are implicated as being signals crucial for social communication within an ant colony.

By functionally characterizing a representative subset of the nine-exon subfamily of *HsOr*s, we found that many responded to long chain CHCs, representing the first fully identified and functionally characterized receptors for these low volatility compounds. In *D. melanogaster*, the response profile of a single receptor can sometimes be mapped to a neuron type by comparing the response profiles of individual endogenous ORNs in a sensillum. This is possible because dipteran sensilla typically contain only 1–3 ORNs and generate action potential spike trains that can be sorted according to their characteristic spike amplitude^27^. In contrast, this is not yet possible in ants because their sensilla basiconica contain scores of ORNs that preclude identifying ORN-specific response profiles^28^. One of the only ways to study the response profiles of individual ant *Ors* therefore would be by deorphanization in a functional expression system like *Drosophila*, as has been previously reported for several other insect receptors, and as we have done here. However, because it is technically not feasible to validate the receptor responses in the endogenous ant neurons, we do not know whether ant-specific factors like sensillar architecture, neuronal structure, odor binding proteins, or other differences could alter the strength of the receptor responses.

### Coding of hydrocarbons by combinations of *HsOrs*

Overall, we generated a data set of 814 hydrocarbon–receptor combinations which revealed that individual receptors respond to small subsets of hydrocarbons, and conversely, individual odorants activate only small subsets of receptors (Fig. 4c). This is consistent with a combinatorial model of hydrocarbon coding, where a repertoire of receptors can detect and discriminate relevant compounds in a large chemical space. Because the response spectrum of each of the characterized HsOrs is narrow, possessing an expanded family of receptors can augment the discriminatory power of the olfactory system. Because none of the receptors tested showed strong responses to hydrocarbons shorter than C28 (Fig. 4c), it is possible that we have not yet identified receptors for shorter chain hydrocarbons, or these are not used as signals in *H. saltator*. It is also possible that receptors from other *Or* subfamilies are responsible for detecting the shorter hydrocarbons. In fact, several other subfamilies besides the nine-exon subfamily are greatly expanded in social Hymenoptera and may also contribute to CHC detection^9^.

We next constructed a 22-dimensional “receptor space” that correlates hydrocarbons with the receptors that they activate, where each axis represented the responses (spikes s^−1^) for one odorant receptor. Using principal components analysis (PCA), we transformed this into a 3-dimensional space represented by the top three principal components (Fig. 4d), in which hydrocarbons that elicited similar patterns of activity across the receptor repertoire mapped closer together, signifying similar perceptual qualities. The shorter chain hydrocarbons of up to C27 (*grey dots*) and the unsaturated hydrocarbons (*dark grey dots*) clustered together because they did not strongly activate any of the tested HsOrs. The methyl-branched compounds (*blue dots*) and ≥C28 straight-chain hydrocarbons (*green dots*) were well separated in this receptor space (Fig. 4d). The single dimethyl branched compound, 13,23-DiMeC37 (*red dot*), mapped to a region of the receptor space that was distinct from the other compounds (Fig. 4d). This may simply represent differential detection of dimethylalkanes from the other three hydrocarbon types, but it is also conceivable that this candidate queen pheromone component is perceived as distinctly different than other CHCs. A similar analysis of cuticular extracts in the 22-dimensional “receptor space” indicated that extracts of males, workers, and gamergates map to distinct, well-separated regions (Fig. 4e). The extracts from different individuals mapped to distinct regions in the receptor space, but grouped to regions according to the three castes (Supplementary Fig. 1). This suggests that the tested set of nine-exon *HsOr*s, that presumably represents only a modest fraction of *H. saltator*’s resolution potential, has the potential to effectively discriminate among castes within a colony.

From the heat map, these nine-exon hydrocarbon receptors seem narrowly tuned, with strong responses to ~1 compound and half-maximal responses to ~2 compounds. This is similar to the pheromone receptors in *D. melanogaster* but quite different from the large numbers of broadly tuned general host-odorant receptors described in *Anopheles gambiae* or *D. melanogaster* ^29–31^. The ability of these ant Ors to discriminate among CHCs with very similar structures might reflect a strong benefit from having finely-tuned behavioural responses to a large variety of nuanced social cues.

## Discussion

The nine-exon subfamily of HsOrs responded to cuticular extracts from several castes, and also to individual CHCs found in extracts of *H. saltator*. Additionally, the predicted queen pheromone component in *H. saltator*, 13,23-DiMeC37, elicited strong responses in HsOr263. It remains to be determined as to how activation of this receptor, and possibly others that remain to be discovered, mediates reproductive division of labour within the colony. Our identification of HsOr263 as a candidate receptor for a queen pheromone component suggests that future studies aimed at unravelling the neurobiological and physiological mechanisms of social organization and division of labour in ants should include a meticulous characterization of the connectivity of olfactory neurons expressing this receptor. While the nine-exon subfamily was targeted in this initial study, several other *Or* gene subfamilies (U, V, E, and L) are also expanded in social insects and these may also be used in perception of CHCs from ants or prey^6–10^.

In summary, the detection of CHCs by combinations of narrowly tuned Ors and the large family of *Or* genes likely underlies the extraordinary ability of ants to differentiate between the structurally-related CHCs that mediate the organization and survival of their complex colonies^32^. This distributed system of detection provides the information input to higher brain centres where the complex CHC signatures associated with eusocial recognition are translated into appropriate behavioural responses. Overall, this work offers a model for the molecular basis of CHC detection in ants, and suggests an important role for the nine-exon *Ors* in the proper organization and functioning of insect societies.

## Methods

### HsOr cloning and UAS fly generation

RNA was extracted from resected and homogenized *H. saltator* worker antennae using TRIzol reagent (Life Technologies, Carlsbad, CA, USA). Total antennal RNA was then oligo(dT)-primed for cDNA synthesis with Superscript II Reverse Transcriptase (Life Technologies). Predicted full-length *HsOr* coding sequences were PCR amplified from antennal cDNA templates and cloned into either pENTR/D-TOPO (Life Technologies) or pATTL entry vectors. *HsOrs* in entry vectors were then sub-cloned into the pUASg.attB (a gift from Konrad Basler, University of Zurich) using LR Clonase II (Life Technologies). These constructs were then coinjected into y, w; attP40 *D. melanogaster* embryos with a phiC31 integrase plasmid (Genetic Services, Inc., Sudbury, MA, USA).

### *Harpegnathos saltator* extract preparation

Whole bodies of freeze-killed gamergates and non-reproductive workers were individually extracted in 500 µl hexane for 5 min and the extracts were analysed by GC/MS using a DB1 column to check for possible contamination or inadequate extraction. Sufficiently clean extracts from 23 gamergates from 19 colonies and 26 non-reproductive workers from 15 colonies were consolidated, and the two consolidated extracts were again analysed by GC/MS. Whole bodies of 50 males originating from 14 colonies were extracted in two batches of 25 individuals in 2 ml hexane for 5 min each before consolidation into one batch. The gamergate extract consisted of 99.1%, non-reproductive worker of 96.7%, and male of 99.9% CHCs, respectively. Other compounds present in the extract were largely short-chain hydrocarbons smaller than pentacosane that do not regularly appear on the worker cuticle. Total cuticular hydrocarbon yield was determined by calibration using tetracosane as an internal standard. Consolidated CHC extracts were then diluted to 0.6 µg/µl in pentane for use in electrophysiology assays.

### Syntheses of CHCs

The syntheses of several unsaturated and branched CHCs in the panel have been previously described^33–35^.

*General*. All reactions were performed under an argon atmosphere with oven-dried glassware. Crude products were purified via flash or vacuum flash column chromatography unless otherwise stated. Yields refer to isolated yields of purified products. Tetrahydrofuran (THF) was distilled from sodium/benzophenone ketyl under an argon atmosphere, and all solvents utilized were Optima grade (Fisher Scientific, Pittsburg, PA, USA). Mass spectra were obtained with a Hewlett-Packard (HP) 6890 GC (Hewlett-Packard, Avondale, PA, USA) interfaced to an HP 5973 mass selective detector, in EI mode (70 eV) with helium as carrier gas. The GC was equipped with a DB17-MS column (25 m × 0.20 mm i.d., 0.33 μm film). ^1^H and ^13^C NMR spectra were recorded with a Varian INOVA-400 (400 and 100.5 MHz, respectively) spectrometer (Palo Alto, CA, USA), as CDCl_3_ solutions. ^1^H NMR chemical shifts are expressed in ppm relative to residual CHCl_3_ (7.27 ppm) and ^13^C NMR chemical shifts are reported relative to CDCl_3_ (77.16 ppm).

**Fig. 5:**
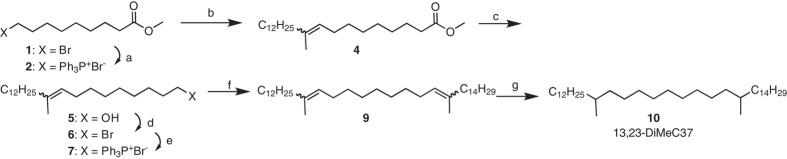
Synthesis of 13,23-dimethylheptatriacontane. a Ph_3_P, acetonitrile, reflux; b NaHMDS, then 2-tetradecanone 3, CH_2_Cl_2_; c LiAlH_4_, THF; d Ph_3_P, CBr_4_, CH_2_Cl_2_; e Ph_3_P, 110 °C; f NaHMDS, then 2-hexadecanone, CH_2_Cl_2_; g 5% Pd/C, H_2_, hexane

*Synthesis of 13,23-dimethylheptatriacontane* ***10***. Figure 5 Triphenylphosphine (1.09 g, 4.15 mmol) and ethyl 9-bromononanoate (1.1. g, 4.15 mmol; TCI Americas, Portland, OR, USA) were refluxed in 10 ml acetonitrile for 36 h. Most of the acetonitrile was removed by rotary evaporation, and the resulting gum was triturated 4 times with ethyl ether. The remaining gum would not crystallize, and so it was taken up in 5 ml CH_2_Cl_2_, and ~2/3 of the solution was transferred to a tared vial. Removal of the solvent gave 1.23 g (2.3 mmol) of the phosphonium salt **2**, which was used without further purification.

The phosphonium salt **2** was taken up in 10 ml CH_2_Cl_2_, cooled to −78 °C under argon, and NaHMDS (2 M in THF) was added dropwise until the solution turned orange, then a further 1.2 ml (2.4 mmol) of NaHMDS solution was added. The resulting solution was stirred 30 min, then 2-tetradecanone **3** (0.3 g, 1.4 mmol) in 5 ml CH_2_Cl_2_ was added dropwise. The resulting solution was warmed to room temp over 4 h, then quenched with 1 M HCl, and the mixture was extracted with hexane. The hexane layer was washed with saturated aqueous NaHCO_3_ solution and brine, then dried and concentrated. The residue was purified by vacuum flash chromatography, eluting with 3% EtOAc in hexane, yielding ethyl 10-methyldocos-9-enoate **4** (0.42 g, 79%) as a 4:3 mixture of *E*-isomers and *Z*-isomers. Isomer 1 MS (*m*/*z*, abundance) 380 (22), 335 (33), 227 (11), 211 (29), 210 (27), 180 (44), 171 (94), 165 (35), 155 (13), 147 (17), 137 (19), 125 (69), 111 (37), 97 (81), 83 (76), 69 (91), 55 (100), 43 (52), 41 (48). Isomer 2: MS (*m*/*z*, abundance) 380 (23), 335 (35), 227 (12), 211 (27), 210 (27), 180 (46), 171 (100), 165 (34), 155 (13), 147 (16), 137 (21), 125 (65), 111 (38), 97 (72), 83 (73), 69 (89), 55 (97), 43 (45), 41 (41).

The mixture of esters **4** in ~2 ml THF was then added to a slurry of LiAlH_4_ (84 mg, 2.2 mmol) in 5 ml THF cooled to –78 °C under argon. The resulting mixture was warmed to room temperature and stirred 2 h, then quenched by sequential dropwise addition of water (88 µl), 20% aqueous NaOH (66 µl), and water (310 µl). After stirring 20 min, the mixture was filtered through a plug of Celite, rinsing well with ether. Concentration yielded 10-methyldocos-9-en-1-ols **5** (0.21 g, 65%, 96% pure by GC) as a 4:3 mixture of isomers, which were used immediately in the next step.

Carbon tetrabromide (232 mg, 0.7 mmol) and triphenylphospine (187 mg, 0.7 mmol) were added to an ice-cooled solution of alcohol **5** (0.21 g, 0.62 mmol) in 5 ml CH_2_Cl_2_, the mixture was stirred 30 min, then the cooling bath was removed and the mixture was stirred a further 3 h. The mixture was then concentrated, and the residue was purified by vacuum flash chromatography, eluting with hexane, yielding 10-methyldocos-9-en-1-yl bromide **6** (237 mg, 0.57 mmol, 92%) as a colourless oil, as a 4:3 misture of isomers. Isomer 1, MS (*m*/*z*, abundance) 402 (10), 400(11), 248 (17), 246 (20), 233 (13), 231 (15), 220 (10), 218 (8), 210 (12), 195 (12), 151 (14), 125 (15), 123 (11), 111 (42), 97 (72), 83 (73), 69 (94), 57 (79), 56 (87), 55 (100), 43 (45), 41 (45). Isomer 2, MS (*m*/*z*, abundance) 402 (9), 400(10), 248 (14), 246 (14), 233 (13), 231 (11), 220 (10), 218 (8), 210 (12), 195 (8), 151 (10), 125 (16), 123 (8), 111 (38), 97 (64), 83 (76), 69 (89), 57 (77), 56 (89), 55 (100), 43 (41), 41 (465).

The mixture of bromides **6** was mixed with triphenylphosphine (152 mg, 0.57 mmol) and heated at 110 °C overnight under Ar. Toluene (5 ml) was then added to the resulting viscous oil, and the mixture was refluxed for 1.5 days, yielding a pale brown solution. The solvent was removed by rotary evaporation, and 10 ml diethyl ether was added to the residue, giving a viscous oil and a cloudy solution, which was removed. A further 10 ml ether was added and the mixture was stirred 1 h, resulting in a very fine pale brown suspension. The suspension was centrifuged, the liquid phase was removed, and the remaining solids were taken up in ~3 ml CH_2_Cl_2_. An aliquot of the solution was transferred to a clean, dry flask, and concentrated to give 160 mg (~0.25 mmol) of the phosphonium salt **7** as a gum.

The gum was taken up in 5 ml CH_2_Cl_2_, the solution was cooled to −78 °C under argon, and NaHMDS (2 M in THF) was added dropwise until the solution turned yellow. An additional 0.13 ml of NaHMDS solution (0.26 mmol) was added, the mixture was stirred 30 min, and then a solution of 2-hexadecanone **8** (60 mg, 0.25 mmol) in 2 ml CH_2_Cl_2_ was added dropwise. The mixture was warmed to room temperature and stirred overnight, then quenched with 1 M HCl, and extracted with hexane. The hexane extract was washed with saturated aqueous NaHCO_3_ solution and brine, then dried and concentrated. The residue was purified by vacuum flash chromatography, eluting with hexane. The resulting clear oil was taken up in 5 ml hexane, 5% Pd on carbon (10 mg) was added, and after flushing with hydrogen, a balloon of hydrogen was attached to the septum-sealed flask. The mixture was stirred 3 h, then filtered through a plug of Celite, rinsing well with hexane. The resulting white semisolid (~80% pure by GC) was recrystallized from a mixture of 5 ml each of hexane and acetone at −20 °C, yielding 25 mg of the desired 13,23-dimethylheptatriacontane as a white solid, >96% pure by GC. The remaining liquor containing the bulk of the product, contaminated with impurities, was not purified further. MS (*m*/*z*, abundance): 533 (4, M^+^-15) 519 (2), 379 (13), 351 (14), 224 (23), 196 (34), 183 (4), 181 (6), 169 (6), 155 (), 141 (11), 127 (15), 113 (19), 99 (27), 85 (65), 71 (82), 57 (100), 43 (44).

**Fig. 6:**
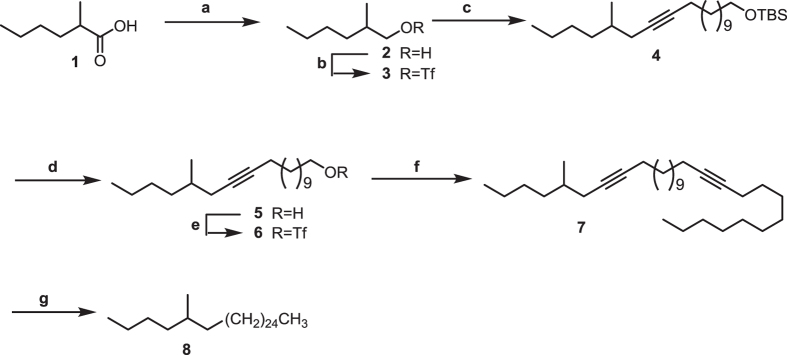
Synthesis of ±-5-methylhentriacontane. a LiAlH_4,_ Et_2_O (95%); b Tf_2_O, pyridine, CH_2_Cl_2_ (quantitative); c *tert*-Butyldimethyl(tridec-12-ynyloxy)silane, *n*-BuLi, THF (74%); d AcCl, dry MeOH (96%); e Tf_2_O, pyridine, CH_2_Cl_2_ (quantitative); f hexadecynyllithium, THF (49%); g 10% Pd/C, H_2_, hexane (~quantitative)

*Synthesis of (±)-5-methylhentriacontane* ***8***. (±)-2-Methylhexanol (**2**). Figure 6 LiAlH_4_ (2.87 g, 75.6 mmol) was suspended in dry Et_2_O (50 ml), the mixture was cooled to 0 °C, and 2-methylhexanoic acid **1** (5.0 g, 37.8 mmol, Sigma-Aldrich, Milwaukee, WI, USA) was added dropwise over 10 min. The resulting mixture was stirred 30 min at 0 °C, then warmed to room temperature and stirred 3 h. The mixture was then cooled to 0 °C and worked up by sequential dropwise addition of 2.9 ml of H_2_O, 2.9 ml of 15% (wt/wt) aqueous NaOH, and 9 ml H_2_O. The resulting suspension was stirred 20 min, then filtered with suction, and the solids were washed with Et_2_O (3 × 75 ml). The combined Et_2_O washes were concentrated and purified by vacuum flash chromatography (EtOAc/hexanes, 1:4) to afford (±)-2-methylhexanol (4.15 g, 95 % yield, 98.7% pure by GC) as a colourless oil. ^1^H NMR (CDCl_3_): ∂ 0.87 (3H, t, 5.7 Hz), 0.89 (3H, d, 6.3 Hz), 1.05 (1H, m), 1.24–1.35 (5H, broad m), 1.62 (1H, m), 1.82 (OH, broad s), 3.35 (1H, dd, *J* = 11.8, 5.8 Hz), 3.48 (1H, dd, *J* = 12.3, 6.8 Hz). ^13^C NMR (CDCl_3_): ∂ 14.1, 17.0, 23.5, 29.8, 33.0, 38.5, 68.5 ppm. EIMS (*m*/*z*, abundance): 115 (5, M^+^-1), 98 (12), 84 (28), 70 (45), 56 (100), 42 (57).

(±)-2-Methylhexan-1-yl triflate (**3**). Pyridine (277 μl, 3.44 mmol) and triflic anhydride (704 μl, 4.1 mmol) were added sequentially to a cold (−10 °C) solution of (±)-2-methylhexan-1-ol **2** (400 mg, 3.44 mmol) in CH_2_Cl_2_ (15 ml). The mixture was stirred for 1 h at −10 °C and then diluted with pentanes (50 ml), warmed to room temperature, and filtered through a plug of silica gel. The filter cake was rinsed with 3:1 hexane:CH_2_Cl_2_ (3 × 50 ml) to ensure that all of the alkyl triflate intermediate was recovered. The filtrates were then combined and concentrated to give triflate (±)-**3** (854 mg, quantitative) as a colourless oil, which was used immediately in the next step.

(±)-*tert*-Butyldimethyl(15-methylnonadec-12-ynyloxy)silane (**4**). *tert*-Butyldimethyl(tridec-12-ynyloxy)silane (1.013 g, 3.27 mmol) was dissolved in dry THF (15 ml), the solution was cooled to −78 °C, and *n*-BuLi (2.2 M, 1.48 ml) was added dropwise. The reaction was stirred at −78 °C for 1 h, then (±)-2-methylhexan-1-yl triflate **3** (854 mg, 3.44 mmol) in 3 ml THF was added by syringe pump over 30 min, and the reaction was warmed −40 °C and stirred for 5 h. The mixture was then quenched with water (20 ml) and extracted with hexane. The hexane extract was washed with brine, dried, and concentrated, and the residue was purified by vacuum flash chromatography (EtOAc/hexanes, 1:5) to afford (±)-*tert*-butyldimethyl(15-methylnonadec-12-ynyloxy)silane (988 mg, 74% yield, 98.3% pure by GC) as a colorless oil. ^1^H NMR (CDCl_3_): ∂ 0.21 (6H, s), 0.89 (3H, t, 6.3 Hz), 0.93 (3H, d, 6.5 Hz), 0.98 (9H, s), 1.2–1.5 (25H, br m), 2.08 (4H, m), 3.38 (2H, t, *J* = 6.3 Hz). ^13^C NMR (CDCl_3_): ∂ -1.9, 14.0, 18.2, 19.5, 26.3, 28.7, 29.5, 30.2, 30.5, 31.3, 33.2, 36.5, 38.0, 64.1, 78.3, 80.1 ppm. EIMS (*m*/*z*, abundance): 351 (35, M^+^-57), 277 (3), 265 (5), 249 (1), 207 (1), 193 (1), 179 (1), 165 (1), 151 (1), 137 (5), 99 (11), 85 (75), 71 (61), 57 (100), 43 (67).

(±)-15-Methylnonadec-12-yn-1-ol (**5**). Acetyl chloride (30 µl, 0.32 mmol) was added to a cooled (0 °C) solution of (±)-*tert*-butyldimethyl(15-methylnonadec-12-ynyloxy)silane **4** (900 mg, 2.15 mmol) in dry MeOH (10 ml). The reaction was stirred for 10 min at 0 °C, then warmed to room temperature for another 20 min. The reaction was then diluted with Et_2_O (25 ml) and quenched with 10% aqueous NaHCO_3_. The layers were separated and the aqueous layer was washed with Et_2_O (2 × 50 ml). The combined organic layers were washed with water (2 × 50 ml) and brine (2 × 50 ml), dried, and concentrated. The residue was purified by vacuum flash chromatography (EtOAc/hexanes, 1:5) to afford (±)-15-methylnonadec-12-yn-1-ol (602 mg, 96% yield, 98.3% pure by GC) as a colourless oil. ^1^H NMR (CDCl_3_): ∂0.88 (3H, t, 6.1 Hz), 0.90 (3H, d, 6.5 Hz), 1.2–1.5 (25H, br m), 1.83 (OH, broad s), 2.08 (4H, m), 3.51 (2H, t, *J* = 6.0 Hz). ^13^C NMR (CDCl_3_): ∂ 14.0, 17.3, 19.2, 26.5, 28.7, 29.7, 30.2, 30.8, 31.3, 33.2, 36.5, 38.0, 62.3, 78.5, 81.1. EIMS (*m*/*z*, abundance): 293 (5, M^+^-1), 276 (3), 263 (1), 249 (1), 235 (1), 209 (5), 195 (2), 171 (2), 151 (1), 137 (5), 123 (11), 112 (14), 99 (23), 85 (76), 71 (68), 57 (100), 43 (77).

(±)-15-methylnonadec-12-yn-1-yl triflate (**6**). Pyridine (41 μl, 0.51 mmol) and triflic anhydride (104 μl, 0.61 mmol) were added sequentially to a cold (−10 °C) solution of (±)-15-methylnonadec-12-yn-1-ol **5** (150 mg, 0.51 mmol) in CH_2_Cl_2_ (5 ml). The mixture was stirred for 1 h at −10 °C, then diluted with hexanes (50 ml), warmed to room temperature, and filtered through a plug of silica gel, rinsing with 3:1 hexane:CH_2_Cl_2_ (3 × 50 ml) to ensure that all of alkyl triflate **6** was recovered. The filtrate was concentrated to give 15-methylnonadec-12-yn-1-yl triflate (±)-**6** (219 mg, quantitative) as a colourless oil, which was used immediately without further purification or characterization.

(±)-5-methylhentriacontane (**8**). *n*-BuLi (2.1 M, 0.42 ml, 0.88 mmol) was added dropwise to a cooled solution (−78 °C) of 1-dodecyne (0.21 ml, 0.97 mmol) in 5 ml dry THF. The mixture was stirred at −78 °C for 1 h, followed by dropwise addition of triflate **6** (0.35 g, 0.81 mmol) in 2 ml THF. The mixture was slowly warmed to room temperature, then quenched with water and extracted with hexane. The hexane extract was washed with brine, dried, and concentrated, and the residue was purified by vacuum flash chromatography (hexane) giving (±)-5-methylhentriaconta-7,20-diyne **(7)** as a colourless oil (170 mg, 49%). The oil was taken up in 2 ml hexane and added to a slurry of 10% palladium on carbon (20 mg, 10 % wt, Signa Aldrich) and K_2_CO_3_ (250 mg, 1.81 mmol) in 5 ml hexane. The heterogenous mixture was stirred for 8 h under a slight positive pressure of H_2,_ then filtered through a plug of silica gel and concentrated. Recrystallization of the residue from acetone (5 ml) at −20 °C gave 170 mg of (±)-5-methylhentriacontane (99% pure by GC), mp 36 °C. ^1^H NMR (CDCl_3_): ∂ 0.87 (3H, d, *J* = 6 Hz), 0.89 (3H, t, *J* = 6.1 Hz), 0.91 (3H, t, *J* = 6.2 Hz), 1.16–1.4 (56H, broad m), 1.53 (1H, m). ^13^C NMR (CDCl_3_): ∂_c_ 11.6, 15.3, 19.4, 21.8, 23.5, 24.7, 25.2 27.6, 29.3, 29.7, 30.0, 30.3, 31.8, 32.6, 34.2, 36.8 ppm. EIMS (*m*/*z*, abundance): 435 (1, M^+^-15), 421 (1), 407 (1), 393 (35), 365 (4), 351 (1), 337 (1), 323 (1), 309 (2), 295 (2), 281 (2), 267 (2), 253 (2), 239 (3), 225 (3), 211 (2), 197 (3), 183 (3), 169 (3), 155 (5), 141 (5), 127 (9), 113 (11), 99 (18), 85 (100), 71 (70), 57 (81), 43 (69).

Synthesis of 13- and 15-methylbranched hydrocarbons. Hexadecyltriphenylphosphonium bromide was purchased from Alfa-Aesar (Ward Hill MA) and tetradecyltriphenylphosphonium bromide was obtained from Lancaster Synthesis (Windham NH). Octadecyltriphenylphosphonium bromide was prepared by refluxing a mixture of triphenylphosphine (26.2 g, 100 mmol) and octadecyl bromide (33.3 g, 100 mmol) in 50 ml toluene for 5 days. The resulting viscous oil was poured into a 1:1 mixture of toluene and hexane, and after stirring 30 min, the resulting solids were collected by vacuum filtration, then pumped under high vacuum (0.05 mm Hg) for 8 h, yielding the desired product as an amorphous white powder (47.5 g, 80%).

A slurry of hexadecyltriphenylphosphonium bromide (1.703 g, 3 mmol) in dry THF (6 ml) was cooled to −10 °C under argon, and lithium diisopropylamide (1.5 M in cyclohexane, 2.1 ml, 3.15 mol) was added over ~10 min. The mixture was stirred 3 h, then cooled to −30 °C, and 2-tetradecanone (0.64 g, 3 mmol) in 1.5 ml THF was added dropwise. The resulting mixture was warmed to room temperature and stirred overnight, then quenched with saturated aqueous NH_4_Cl solution. The organic layer was separated, and the aqueous phase was extracted with ethyl ether (3 × 20 ml). The combined organic layers were washed with water and brine, dried, and concentrated. The residue was purified by vacuum flash chromatography, eluting with hexane, yielding 13-methyl-13*E*/*Z*-nonacosene (0.55 g, 44% yield), in a 55/45 ratio of geometric isomers. MS (*m*/*z*, abundance): 420 (M^+^, 8), 266 (7), 251 (7), 210 (9), 195 (11), 181 (3), 167 (5), 153 (5), 139 (10), 125 (19), 111 (38), 97 (59), 83 (53), 69 (68), 57 (100), 55 (86), 43 (84), 41 (55).

The mixture of alkene isomers (5.44 g, 1.29 mmol) was dissolved in 10 ml hexane, 5% Pd on carbon catalyst was added (80 mg), and the septum sealed flask was fitted with a balloon of hydrogen. After flushing with hydrogen, the mixture was stirred overnight, then filtered through a plug of Celite, rinsing with hexane. Removal of the solvent by rotary evaporation yielded (±)-13-methylnonacosane (0.53 g, 96%) as a low-melting white solid. ^1^H NMR: *δ* 0.83 (d, 3H, *J* = 6.4 Hz), 0.88 (t, 6H, *J* = 6.8 Hz), 1.00–1.14 (m, 2H), 1.18–1.40 (m, 51H). ^13^C NMR: *δ* 14.35, 19.96, 22.93, 27.32, 29.60, 29.89, 29.94, 29.97, 30.27, 32.16, 32.98, 37.33. MS (*m*/*z*, abundance): 407 (M-15) (2), 393 (1), 253 (10), 252 (14), 224 (4), 197 (12), 196 (23), 183 (2), 168 (5), 155 (4), 141 (7), 127 (10), 113 (13), 99 (20), 85 (58), 71 (77), 57 (100), 43 (60).

In similar fashion, reaction between 2-tetradecanone and octadecyltriphenylphosphonium bromide gave 13-methyl-13*E*/*Z*-hentriacontene in 39% yield, as a ~ 55/45 mixture of geometric isomers. MS (*m*/*z*, abundance): 448 (M^+^, 6), 294 (7), 279 (5), 266 (4), 210 (7), 195 (11), 182 (5), 167 (4), 153 (5), 139 (10), 125 (18), 111 (36), 97 (58), 83 (52), 69 (63), 57 (100), 55 (78), 43 (82), 41 (47). Hydrogenation then gave (±)-13-methyluntriacontane in 97% yield as a white solid. ^1^H NMR: *δ* 0.83 (d, 3H, *J* = 6.4 Hz), 0.88 (t, 6H, *J* = 6.8 Hz), 1.01–1.14 (m, 2H), 1.18–1.38 (m, 55H). ^13^C NMR *δ* 14.35, 19.96, 22.93, 27.32, 29.60, 29.89, 29.93, 29.96, 30.26, 32.16, 32.97, 37.32. MS (*m*/*z*, abundance): 435 (M-15) (1), 281 (7), 280 (8), 252 (2), 197 (6), 196 (11), 183 (2), 169 (3), 168 (3), 155 (4), 141 (8), 127 (9), 113 (12), 99 (18), 85 (44), 71(69), 57 (100), 43 (72).

15-Methyl-15E/Z-nonacosene was obtained in 46% yield from 2-hexadecanone and tetradecyltriphenylphosphonium bromide. MS (*m*/*z*, abundance): 420 (M^+^, 8), 238 (13), 223 (17), 182 (9), 167 (3), 153 (8), 139 (11), 125 (21), 111 (43), 97 (63), 83 (58), 69 (69), 57 (100), 55 (89), 43 (83), 41 (58). Hydrogenation gave 15-methylnonacosane in 94% yield as a white solid. ^1^H NMR: *δ* 0.83 (d, 3H, *J* = 6.4 Hz), 0.88 (t, 6H, *J* = 6.6 Hz), 1.00–1.15 (m, 2H), 1.16–1.40 (m, 51H).^13^C NMR: *δ* 14.35, 19.96, 22.93, 27.32, 29.60, 29.90, 29.94, 29.97, 30.27, 32.16, 32.98, 37.33. MS (*m/z*, abundance): 407 (M-15) (2), 225 (21), 224 (35), 207 (5), 196 (8), 169 (3), 155 (5), 141 (8), 127 (9), 113 (13), 99 (21), 85 (59), 71(76), 57 (100), 43 (62).

15-methyl-15E/Z-hentriacontene was obtained in 52% yield from 2-hexadecanone and hexadecyltriphenylphosphonium bromide. MS (*m*/*z*, abundance): 448 (M^+^, 6), 266 (7), 251 (8), 238 (10), 223 (8), 210 (7), 182 (5), 167 (5), 153 (7), 139 (12), 125 (19), 111 (39), 97 (58), 83 (53), 69 (63), 57 (100), 55 (80), 43 (87), 41 (50). Hydrogenation yielded (±)-15-methylhentriacontane in 95% yield as a white solid. ^1^H NMR: *δ* 0.83 (d, 3H, *J* = 6.6 Hz), 0.88 (t, 6H, *J* = 7.0 Hz), 1.01–1.14 (m, 2H), 1.18–1.38 (m, 55H). ^13^C NMR: *δ* 14.35, 19.96, 22.92, 27.31, 29.60, 28.89, 29.93, 29.96, 30.26, 32.15, 32.97, 37.32. MS (*m*/*z*, abundance): 435 (M-15) (2), 253 (6), 252 (9), 225 (6), 224 (12), 183 (3), 169 (4), 155 (5), 141 (7), 127 (8), 113 (12), 99 (19), 85 (46), 71(67), 57 (100), 43 (76).

### Electrophysiology

For all single-sensillum recordings, flies were assayed 4–6 days post eclosion. In experiments using the *Orco-GAL4* driver, experimental fly genotypes were *w* ^*+*^ *, UAS-HsOr; w* ^*+*^, *Orco-GAL4*, and control flies were +; *w* ^*+*^, *Orco-GAL4*. For empty neuron experiments, flies were *Δhalo; Or22a-GAL4/UAS-HsOr263*. Flies were mounted and extracellular recordings from a given sensillum were performed as previously described^25^. Trials were randomized within the constraint of fly line availability.

Sensillum type was confirmed using a six-odorant panel consisting of paraffin oil, or 2-heptanone, ethyl acetate, geranyl acetate, *(E*)-2-hexenal, and racemic 1-octen-3-ol dissolved in paraffin oil. Each odorant was diluted 100-fold in paraffin oil and 20 µl of each solution was loaded into a Pasteur pipette delivery cartridge. For hydrocarbons, each compound was diluted to 10 micromolar in neat pentane and 20 nmol (2 µl) were applied to each delivery cartridge (~3 cm from open end of Pasteur pipette). Each hydrocarbon cartridge was then heated for ~1 s (avg temp. 112.5 ± 2.8 °C, *n* = 10) with a handheld butane torch before being puffed over the fly antenna for 1 s in 6 ml of humidified air. Cartridges containing ant cuticular extracts were loaded with a normalized dose of 1.2 µg total hydrocarbon and heated in a similar fashion before application to the fly antennal preparation. Control cartridges were loaded with pentane only.

Spike frequencies were blindly and manually analysed. Spikes were counted in a 200 ms window between 0.4 and 0.6 s of the 1 s stimulus, and the Δspikes/sec was calculated by subtracting both the pre-stimulus spike frequency and then the response of the solvent (pentane) spike frequencies.

### Chemical space analysis

PCA analysis of Or responses to stimuli was performed using JMP software (SAS).

### Data availability

All datasets are available from the authors upon request.

## Electronic supplementary material

Supplementary Information
