# Supplementary material for: Specialized odorant receptors in social insects that detect cuticular hydrocarbon cues and candidate pheromones
Source: Nat Commun. 2017 Aug 17;8:297. doi: 10.1038/s41467-017-00099-1 (PMC5561057; doi:10.1038/s41467-017-00099-1)
Supplement: Supplementary file 1 — Supplementary Information [file 41467_2017_99_MOESM1_ESM.pdf]

File Name: Supplementary Information
Description: Supplementary Figure.

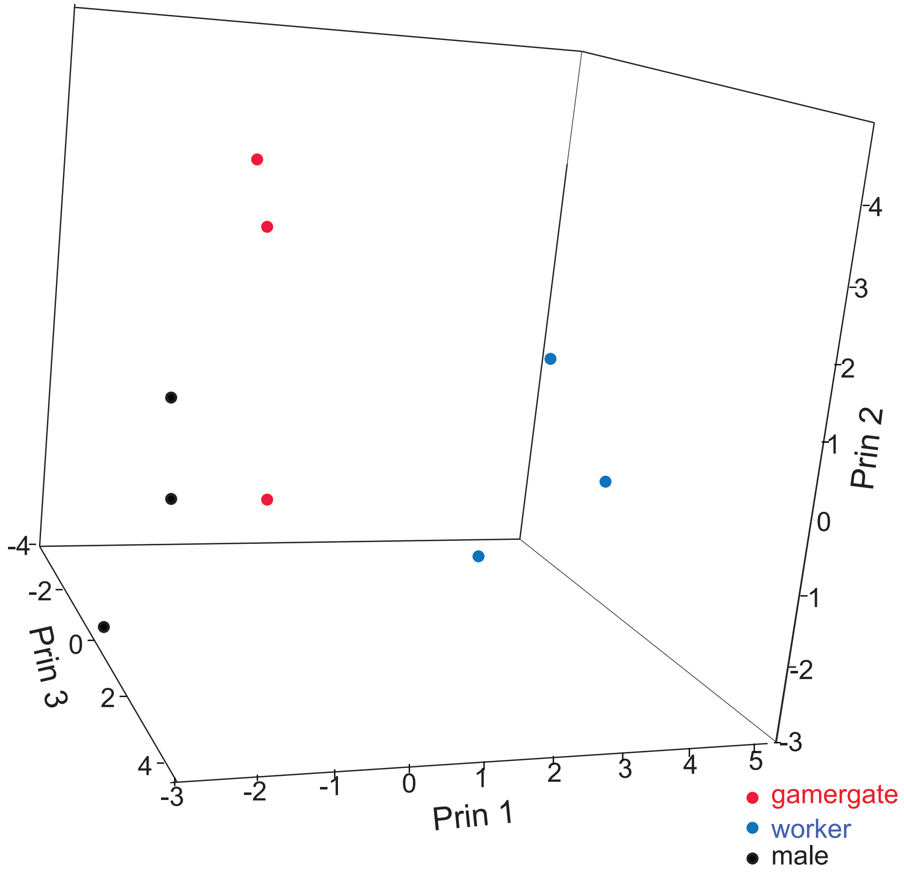

Supplementary Figure 1

The first three principal components of a receptor activity-based space of cuticular extracts from three individuals each of the males, gamergates, and workers.
